# Uncovering the complex regulatory network of spring bud sprouting in tea plants: insights from metabolic, hormonal, and oxidative stress pathways

**DOI:** 10.3389/fpls.2023.1263606

**Published:** 2023-10-23

**Authors:** Junwei Tang, Yao Chen, Chao Huang, Congcong Li, Yue Feng, Haoqian Wang, Changqing Ding, Nana Li, Lu Wang, Jianming Zeng, Yajun Yang, Xinyuan Hao, Xinchao Wang

**Affiliations:** ^1^Key Laboratory of Biology, Genetics and Breeding of Special Economic Animals and Plants, Ministry of Agriculture and Rural Affairs/National Center for Tea Plant Improvement, Tea Research Institute, Chinese Academy of Agricultural Sciences, Hangzhou, China; ^2^Zhejiang Provincial Seed Management Station, Hangzhou, China

**Keywords:** bud sprouting, phenological period, molecular basis, metabolic pathways, plant hormones

## Abstract

The sprouting process of tea buds is an essential determinant of tea quality and taste, thus profoundly impacting the tea industry. Buds spring sprouting is also a crucial biological process adapting to external environment for tea plants and regulated by complex transcriptional and metabolic networks. This study aimed to investigate the molecular basis of bud sprouting in tea plants firstly based on the comparisons of metabolic and transcriptional profiles of buds at different developmental stages. Results notably highlighted several essential processes involved in bud sprouting regulation, including the interaction of plant hormones, glucose metabolism, and reactive oxygen species scavenging. Particularly prior to bud sprouting, the accumulation of soluble sugar reserves and moderate oxidative stress may have served as crucial components facilitating the transition from dormancy to active growth in buds. Following the onset of sprouting, zeatin served as the central component in a multifaceted regulatory mechanism of plant hormones that activates a range of growth–related factors, ultimately leading to the promotion of bud growth. This process was accompanied by significant carbohydrate consumption. Moreover, related key genes and metabolites were further verified during the entire overwintering bud development or sprouting processes. A schematic diagram involving the regulatory mechanism of bud sprouting was ultimately proposed, which provides fundamental insights into the complex interactions involved in tea buds.

## 1 Introduction

Bud sprouting is a critical biological process that marks the beginning of a new life cycle for perennial plants. In the case of tea plant (*Camellia sinensis* (L.) O. Kuntze), where spring shoot sprouting serves as a primary agricultural output, the timing of early and late bud sprouting has a considerable impact on the economic value of tea. Harvesting tender tea buds during early spring is crucial in producing high–grade tea, making tender tea buds a primary agricultural output (Zhao et al., 2021). Tea cultivars that exhibit early and late bud sprouting can display a difference of over one month in their sprouting periods, even when exposed to the same environmental conditions. This variability in sprouting periods provides an excellent opportunity to study the regulatory mechanisms of perennial woody bud sprouting by using tea plant material as a study model. Bud sprouting is a complex process influenced by various internal and external factors. The release of dormancy, the maturity of bud development, the sufficiency of nutrient accumulation, and the alleviation of other limiting factors are internal factors that affect bud sprouting. External factors include changes in photoperiod, temperature, light hours, and agricultural practices (Horvath et al., 2003). Although several physiological and biochemical changes have been documented to occur before and after sprouting (Meier et al., 2012; Sheikh et al., 2022), a comprehensive understanding of the changes occurring at the transcriptional and metabolic levels remains elusive.

In response to sustained low temperatures during winter, plants undergo dormancy to survive. The release of dormancy is a prerequisite for successful sprouting. Bud dormancy release is a multifaceted process that is subject to regulation by many influencing factors, in which phytohormones are crucial signaling modulators (Pan et al., 2021). Abscisic acid (ABA) plays a critical role in regulating bud dormancy, as it integrates environmental factors and metabolites to control the timing of dormancy induction and release (Pan et al., 2021). Decreased ABA levels trigger the release of dormancy, leading to plant growth and development. In grape buds, the ABA catabolic enzyme VvA8H–CYP707A4 accumulates during the release phase, and its overexpression promotes bud release by increasing ABA catabolism (Zheng et al., 2018). Gibberellin (GA) plays a positive role in the release of bud dormancy. In *Jatropha curcas*, GA has been proven to inhibit the expression of *BRC1* and *BRC2*, which perform key functions in maintaining the bud dormancy state (Ni et al., 2015). Similarly, cytokinins (CKs) are also considered key regulators of dormancy release (Liu and Sherif, 2019), and a model of CKs involvement in bud activation has been proposed for apple trees (Li et al., 2018). In this model, the upregulation of CKs signalling stimulates auxin transport and the expression of several axillary meristem activity–related genes, enhancing the growth and development ability of buds in the dormant release stage.

The ability of perennials woody plants to resume growth in the spring season is largely reliant upon their capacity to mobilize sugars and starch, with sugar reserves also being critical for winter survival (Tixier et al., 2019). Particularly glucose metabolism performs a crucial function in the activation of target–of–rapamycin (TOR) activity (Xiong et al., 2013), which in turn affects the growth and developmental processes of plants. Otherwise, the exchange of substances between cells is more frequent during the dormant bud release stage (Cooke et al., 2012). However, when dormant buds are formed, the plasmodesmata become obstructed by blockages consisting of a caloric and proteinaceous substance called the dormancy checkpoint complex, resulting in mutual isolation of cells (Rinne et al., 2011). Cold treatment induces the release of dormant buds, which appears to be associated with the regulation of plasmodesmatal occlusion and opening by intracellular enzymes (Rinne et al., 2001). Furthermore, bud dormancy release may also be modulated by epigenetic mechanisms (Rios et al., 2014). De La Fuente et al., detected significant enrichment of dormancy–related transcription factors such as *DAM* family genes in H3K27me3 histones in peach tree dormancy–releasing buds, indicating that bud dormancy is regulated by methylation (De La Fuente et al., 2015). During dormancy, reactive oxygen species (ROS) tend to accumulate, causing harm to cellular structures. The antioxidant enzyme system, including enzymes like superoxide dismutase (SOD), catalase (CAT), peroxidase (POD), and others, acts as a crucial defense mechanism to eliminate ROS. These enzymes work together to neutralize harmful substances and maintain cellular redox balance (Beauvieux et al., 2018).

Research on tea plants has revealed that early sprouting is associated with a shorter period of profound dormancy, and tea cultivars with shorter dormancy periods have shown increased induction of antioxidative enzymes (Vyas et al., 2007). The identification and characterization of genes related to plant hormones such as auxin, GA, and ABA during the dormancy transition in tea plants highlight the crucial role of these hormones in regulating bud dormancy (Yue et al., 2018; Hao et al., 2019). During the dormancy transition, there are variations in the extent of material exchange. Previous research indicates that overwintering buds exchange substances in various tissues during the initial stage of dormancy establishment and release in tea plants, with a pattern of exchange that transitions from intense to weak and then back to intense (Tang et al., 2017). Changes in substance exchange may trigger molecular and physiological events that promote bud growth and release from dormancy (Cooke et al., 2012), but the mechanism is not fully understood.

The physiological processes of bud reactive growth from dormancy release are significantly influenced by photoperiod and temperature. Long exposure to low temperatures can prompt the release of dormant buds (Singh et al., 2017). Low temperatures can also perturb the circadian rhythms of plants, which are governed by genes such as *LHY* and *TOC1* (Ramos et al., 2005). The duration and appropriateness of the growing season of deciduous trees and shrubs are often quantified by cumulative effective temperature (CET). CET is closely related to plant dormancy release time and the emergence of new growth (McKown et al., 2018). In *Populus trichocarpa*, the release of buds from dormancy gradually advances with an increase in cumulative effective temperature, and there are variances in release time among different genotypes. Similarly, photoperiod and temperature significantly influence the establishment and transition of dormancy in tea plants (Hao et al., 2018b).

At the molecular level, the regulatory mechanisms that control dormancy release and sprouting onset in plants are closely associated with the expression of specific genes. Previous research has identified the role of Early Bud–Break 1 (*EBB1*) in initiating bud dormancy release by promoting the growth of meristems during the early stages of bud development (Yordanov et al., 2014). Studies on Japanese pear have shown that upregulation of *PpEBB* expression during the early stages of bud dormancy release acts on the promoters of cell cycle (*CYCD*) genes (Anh Tuan et al., 2016). Additionally, Liu et al., demonstrated that heterologous expression of agave *AaRVE1* in *poplar* can both delay bud dormancy in autumn and accelerate bud sprouting in spring (Liu et al., 2022a). Tan et al., used linkage mapping to investigate the tea bud sprouting index by mapping quantitative trait loci (QTLs). The results revealed that the tea bud sprouting index was associated with two major QTLs and three secondary QTLs (Tan et al., 2022). Furthermore, 22 critical candidate genes were identified within the QTL confidence interval as being involved in essential processes such as plant hormone signal transduction, stress response, and DNA methylation. This suggests that the mechanisms involved in tea plant sprouting are complex.

Multi-omics methods offer powerful insights into plant biology, enabling a comprehensive understanding of genetic and metabolic processes. Currently, significant insights have been garnered through research on the transcriptional and metabolic regulation of bud sprouting in perennial woody plants. Following manual removal of pear leaves, auxin enrichment at the stem base of flower buds promoted their sprouting. Transcriptome analysis further revealed significant changes in auxin metabolism, transport, and signal transduction pathways after leaf removal (Wei et al., 2022). RNA-seq analysis on dormant peach buds with or without ethephon treatment, indicated that ethephon triggered a stress response during natural dormancy, resulting in a delay in biological processes of cell division and intercellular transport (Liu et al., 2022b). Transcriptomic analysis of the underground renewal buds in penoy highlighted the connections between gene transcriptional regulation and metabolic changes of hormones and ROS during dormancy transition (Zhang et al., 2015). Lately, transcriptional and metabolic analysis is being applied to elucidate the molecular processes of bud dormancy and sprouting in tea plants (Chen et al., 2023). Though previous studies offered a broader insight on dormancy-to-growth transition, the understanding of varietal differences in sprouting time remains limited in tea and other plants.

This study aims to investigate the molecular basis of bud sprouting in tea plants and to gain comprehensive insights into the mechanism of spring sprouting in perennial plants. Specifically, we verify the metabolic and transcriptional profiles of buds in differential developmental stages during sprouting period, companied by investigating key genes and metabolite pathways associated with bud sprouting. Ultimately, we sought to plot a regulatory mechanism for tea bud sprouting based on the integrated results to gain a deeper understanding.

## 2 Materials and methods

### 2.1 Experimental materials and sampling

Experimental materials were planted in the germplasm resource garden located at the Tea Research Institute of the Chinese Academy of Agricultural Sciences (Hangzhou, Zhejiang Province, China (30°N18′, 120°E08′)). In the same plot, twelve lines were selected and sampled on April 13, 2022, in which six lines of ‘Yuqilin’, ‘Leibo 23’, ‘Guizhoudamaiya’, ‘Ankangzhong’, ‘Shubei’, ‘Wuyi 90’ were in bud break stage (respectively named BBS-1, BBS-2,BBS-3, BBS-4, BBS-5, BBS-6) and six lines of ‘Wuyi 43’, ‘Yunhun’, ‘Wuyi 83’, ‘Mandihong’, ‘Magucha’, ‘Ningzhou 6’ were in bud dormancy stage (respectively named BDS-1, BDS-2,BDS-3, BDS-4, BDS-5, BDS-6). The axillary buds of each line were collected for gene expression, transcriptional, and metabolism analyses. A portion of the bud samples were embedded in glutaraldehyde for histological analysis. Meanwhile, the sprouting time was recorded for each line until bud sprouting (one bud and a leaf). For bud dormancy and release study, overwintering buds from three cultivars (‘Longjing 43’ (LJ43), extra–early sprouting cultivar; ‘Biyun’ (BY), intermediate sprouting cultivar; ‘Zhenghe Dabaicha’ (ZHDBC), late sprouting cultivar) were collected from November 2021 to March 2022. For LJ43, overwintering buds were collected during the dormancy formation period (DFP) in November, deep dormancy period (DDP) in December and January, dormancy release period (DRP) in February, and bud sprouting period (SP) in March (Ni et al., 2017). For bud sprouting study, buds from LJ43 were collected at the early sprouting phase (S1, Mar 08), sprouting phase (S2, Mar 10), “fish leaf” phase (S3, Mar12), early one bud and a leaf phase (S4, Mar 14), and one bud and a leaf phase (S5, Mar 16) as previously described (Hao et al., 2018a). Three biological replicates were performed for each tea line, with at least 80 axillary buds per replicate. All samples were quickly frozen in liquid nitrogen and stored at –80°C.

### 2.2 Haematoxylin–eosin staining

The technique for bud sectioning primarily follows the approach described by Feldman, with some modifications (Feldman and Wolfe, 2014). In brief, after dewaxing the paraffin sections, the sections were stained with haematoxylin and eosin, followed by dehydration and sealing with neutral gum. The mounted slides were observed and photographed using a Nikon Eclipse CI microscope and Nikon FI3 (Nikon, Japan). K–Viewer software was used to image at 4× magnification, and the resulting images were formatted in Adobe Photoshop 2018.

### 2.3 Transcriptomics analysis

The TIANGEN RNA extraction kit (Tiangen, China) was used to extract total RNA, followed by mRNA enrichment using oligo (dT) beads. The NEBNext^®^ Ultra™ RNA Library Prep Kit for Illumina^®^ was used for library preparation, followed by sequencing on the Illumina NovaSeq 6000 (Illumina, USA) platform with paired–end reads of 150 base pairs. The quality and quantity of total RNA were assessed using the Agilent 2100 bioanalyzer.

Fastp (version 0.19.7) was used for quality filtering of raw data, followed by alignment against a reference genome (*Camellia sinensis* (L.) O. Kuntze) obtained from an online website (https://bigd.big.ac.cn/search/?dbId=gwh&q=GWHACFB00000000) (Wang et al., 2020). HISAT2 (version 2.0.5) was used for alignment, while gene expression levels were quantified using featureCounts (version 1.5.0–p3), as described in previous literature (Trapnell et al., 2010). Differential expression analysis was performed using the DESeq2 (version 1.20.0) algorithm (Anders et al., 2015), with criteria for differential expression defined as |log2(FoldChange)| >=1 and *P* value < 0.05. The clusterProfiler R package was used to implement Kyoto Encyclopedia of Genes and Genomes (KEGG) and Gene Ontology (GO) analyses (Wu et al., 2021).

### 2.4 Quasi–targeted metabolomics analysis

Samples were ground in liquid nitrogen, 100 mg was added to prechilled 80% methanol, vortexed, and incubated on ice for 5 min. After centrifugation, a portion of the supernatant was mixed with LC–MS grade water and diluted to a final concentration of 53% methanol. This diluted sample was then centrifuged again and injected into the LC–MS/MS system for analysis. Quality control samples were created by mixing equivalent volumes of each experimental sample. Blank control samples were prepared using 53% aqueous methanol and underwent the same pretreatment as the experimental samples. LC–MS/MS analysis was conducted using an ExionLC™ AD system (SCIEX) and a QTRAP^®^ 6500+ mass spectrometer (SCIEX). Samples were separated on an Xselect HSS T3 (2.1×150 mm, 2.5 μm) column using a 20–minute linear gradient and analyzed in both positive and negative polarity modes with eluent A (0.1% formic acid–water) and eluent B (0.1% formic acid–acetonitrile).

The analysis of metabolites was performed using an in–house–developed database (nonoDB) and multiple reaction monitoring (MRM). Chromatographic peaks were subjected to integration and correction procedures using SCIEX software, with final data being analyzed and visualized for further analysis (He et al., 2021). The Kyoto Encyclopedia of Genes and Genomes (KEGG) database (https://www.genome.jp/kegg/pathway.html) was utilized for metabolite annotation. MetaX software was utilized to conduct principal component analysis (Wen et al., 2017). Metabolites exhibiting differential expression were identified based on the following criteria: VIP (variable importance in projection) score >1, a *P* value <0.05, and a fold change of either ≥2 or ≤0.5.

### 2.5 Gene–metabolite correlation analysis

The R statistical analysis program (Zhu et al., 2019) was used to calculate the correlation coefficient of metabolites with genes (*P*<0.05). Gene–metabolite pairs with a Pearson correlation coefficient >0.6 were used to build a transcription–metabolite network, which was visualized using Cytoscape software (Cline et al., 2007).

### 2.6 qRT–PCR validation

Total RNA was extracted from 0.1 g tissue samples using the RNAprep Pure Plant Plus Kit (Tiangen, China) and then treated with RNase–free DNase I (Takara, Japan) to remove residual genomic DNA. cDNA was synthesized using the PrimerScript RT reagent kit (Takara, Japan). Quantitative real–time PCR (qRT–PCR) was performed using SYBR Green I Master Mix on a LightCycler 480 Real–time PCR system (Roche, USA), with *CsPTB* used as the internal reference (Hao et al., 2014). The primer sequences used for qRT–PCR are listed in **Table S1** (**Supplementary Table 1**). The reaction setup, program, and data analysis were performed as previously described (Hao et al., 2014).

### 2.7 Determination of antioxidant enzyme activity and antioxidant content

The activities of SOD, POD, CAT, GR, and GST, as well as the levels of GSSG, GSH and H_2_O_2,_ were estimated by the corresponding reagent kits: Superoxide dismutase (SOD) test kit (SOD–1–W), Peroxidase (POD) assay kit (POD–1–Y), Catalase (CAT) detection kit (CAT–1–Y), glutathione reductase (GR) test kit (GR–1–W), glutathione S–transferase (GST) assay kit (GST–1–W), glutathione disulfide (GSSG) detection kit (GSSG–1–W), reduced glutathione (GSH) test kit (GSH–1–W) and hydrogen peroxide (H_2_O_2_) assay kit (H_2_O_2_–1–Y) (Suzhou Comin Biotechnology Co. Ltd, Suzhou, China), following the manufacturer’s instructions. Approximately 0.1 g sample previously ground with 1 ml reagent one (SOD, POD and CAT were mixed with reagent one, while H_2_O_2_ was mixed with acetone) in an ice bath were poured into a 2 ml tube. The tubes were centrifuged at 8,000×g for 10 min (or 15 min for GR) at 4°C, and the supernatants were used for the assay.

The BCA protein assay (Suzhou Comin Biotechnology Co. Ltd, Suzhou, China) was used to quantify soluble proteins in the samples, and the activity of antioxidant enzymes, including GR, GST, SOD, POD, and CAT, was then calculated.

### 2.8 Sugar detection

The concentrations of glucose, sucrose, and soluble sugars were determined using assay kits (glucose assay kit, PT–1–Y; sucrose assay kit, ZHT–1–Y; soluble sugar assay kit, KT–1–Y) from Suzhou Comin Biotechnology Co. Ltd, Suzhou, China, following the manufacturer’s instructions. For glucose detection, samples (0.1 g) were mixed with distilled water, heated in a 95°C water bath, and centrifuged. The supernatant was collected for assay. For sucrose detection, samples (0.1 g) were ground and mixed with extract. The mixture was then incubated at 80°C, followed by decolorization with reagent five and centrifugation, and the supernatant was collected for analysis. The soluble sugar concentration was determined using anthrone colorimetry. Samples (0.1 g) were mixed with distilled water, heated at 95°C, and centrifuged. The supernatant was transferred to 10 mL tubes and adjusted to a final volume for further measurement.

The trehalose 6–phosphate assay was conducted using the Plant Trehalose–6–phosphate (T6P) ELISA kit (YLS–3921, Yilaisa Biotechnology, Jiangsu, China). Briefly, 10 μl of the sample was mixed with sample diluent and HRP–coupled reagent. After incubating at 37°C for 60 min, chromogen solutions A and B were added and incubated for 15 min at 37°C in the dark. The reaction was stopped by adding 15 μl of stop solution for further testing.

### 2.9 Determination of plant hormone concentrations

The detection of plant hormones primarily followed the approach described by Mikiko (Kojima et al., 2009), with some modifications. Briefly, sample extraction was performed using acetonitrile, while the standard solution was prepared using internal standards for each hormone, including GA3, ABA, zeatin, and indole–3–acetic acid (IAA), dissolved in a methanol solution. After impurities were removed using C18 and GCB QuECherS sorbent materials (Shanghai ANPEL), HPLC–MS/MS analysis was conducted using an AGLIENT1290 HPLC system coupled with a SCIEX–6500Qtrap mass spectrometer. The liquid chromatography column utilized was a Poroshell 120 SB–C18 reverse–phase chromatography column (2.1×150, 2.7 μm), and gradient elution was achieved by increasing the percentage from 20% to 80% over 9 minutes, followed by a reduction back to 20%. The monitoring was carried out in ESI positive and negative ion modes, with MRM serving as the scan type.

### 2.10 Statistical analysis

The data were analysed using SPSS Statistics software (version 26) with either Student’s *t* test or one–way ANOVA. The results were expressed as the means ± SE and conducted with at least three biological replications. Statistical significance was set at *P* < 0.05. Heatmaps, column charts, and GO analysis charts were generated using TBtool (https://github.com/CJ-Chen/TBtools), GraphPad Prism 8, and an online website (https://www.chiplot.online/), respectively. Mechanical drawings were created with Adobe Illustrator 2021, and image analysis was performed using Adobe PhotoShop 2018.

## 3 Results

### 3.1 Investigation of tea axillary bud phenotype, structure and response to temperature

**Figure 1:**
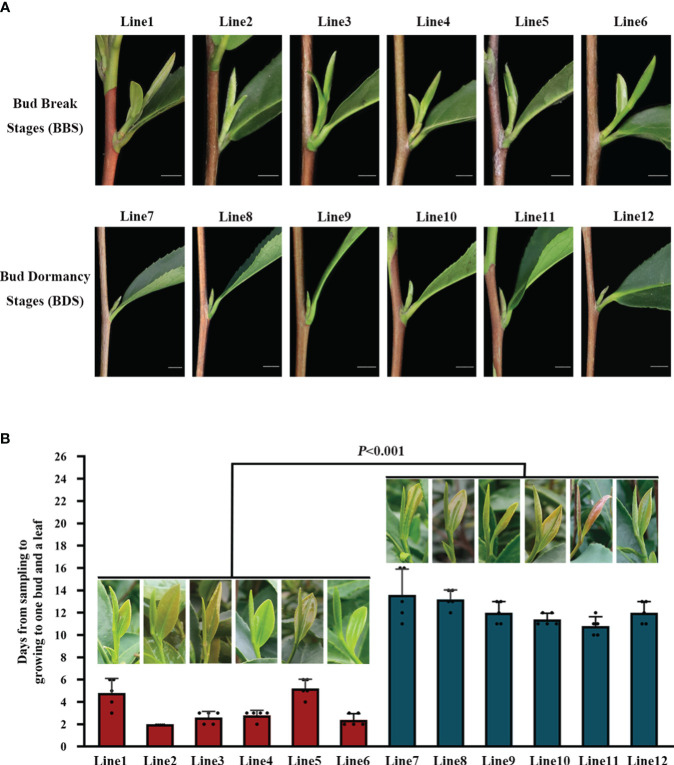
Comparison of phenotypic traits in different bud sprouting stages **(A)**. Timing of bud growth to one bud and a leaf in different bud sprouting states **(B)**. Values represent the means of five replicates ± SE (one-way ANOVA).

**Figure 2:**
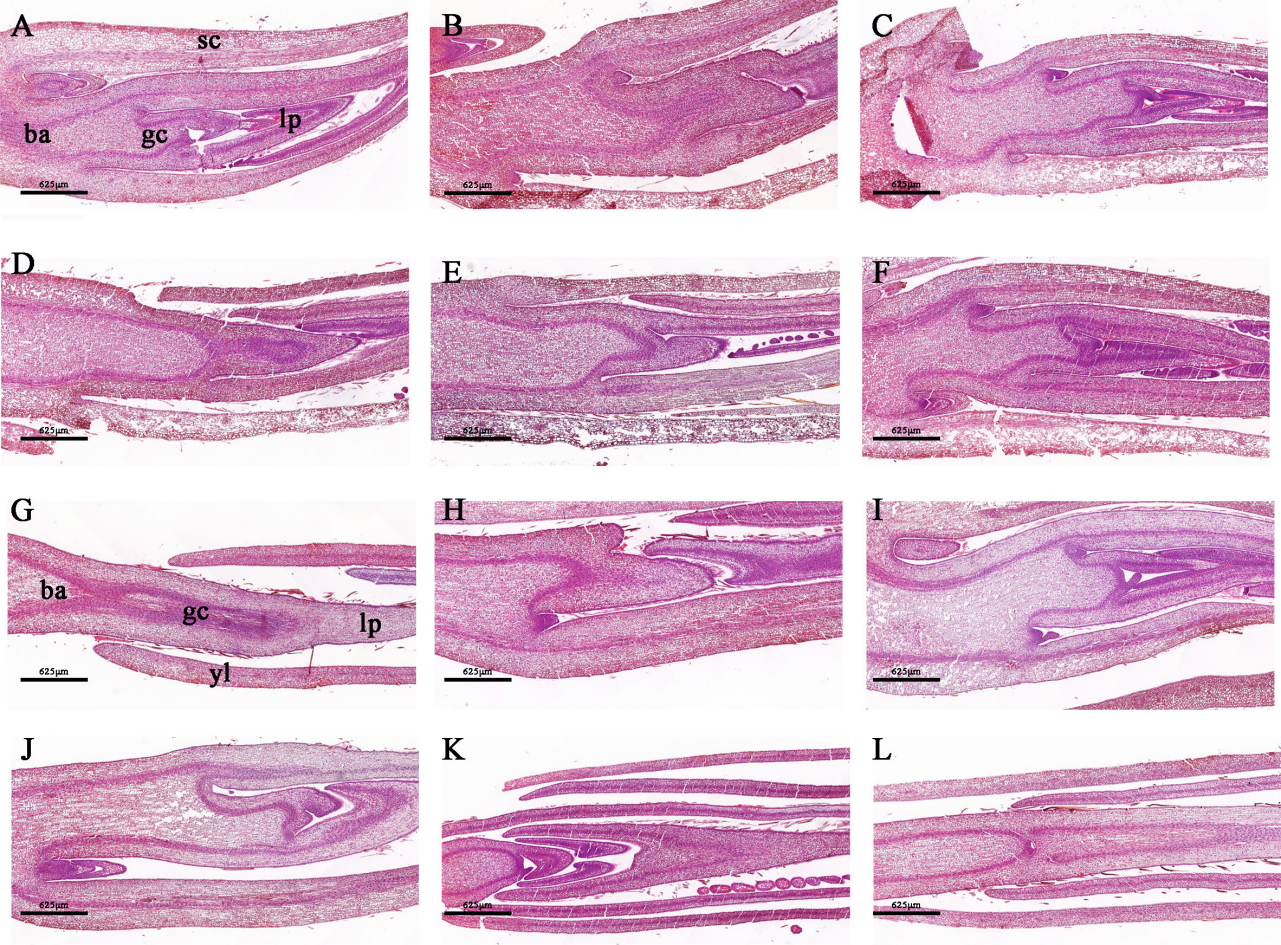
Morphologically stained tissue sections between the BDS **(A–F)** and BBS **(G–L)**. **(A, G)** ba, bud axis; gc, growth cone; lp, leaf primordium; sc, scale; yl, young leaves.

To capture a comprehensive overview of the climatic conditions preceding the sampling, we meticulously recorded daily measurements of the maximum, average, and minimum temperatures during the one–month period prior to harvest (**Supplementary Figure 1**). Our records revealed that over 50% of the days during this period exhibited a maximum temperature exceeding 13°C, which highlights the favorable overall environmental conditions for tea plants sprouting. In an investigation conducted under natural conditions, distinct phenotypic characteristics were observed in different bud sprouting stages (**Figure 1A**). The development from BBS to one bud and a leaf stage took about 4 days on average. In contrast, the development from BDS to one bud and a leaf stage took an average of 13 days (**Figure 1B**). Furthermore, the sampled bud tissue was sectioned for histological analysis, which unveiled typical internal architecture, including young leaves, leaf primordium, bud axis, and growth cone in both groups (**Figure 2**). Specifically, break bud demonstrated looser structures and an increased proportion of young leaves compared to dormancy bud, indicated notable distinctions in their internal physiological states.

### 3.2 Transcriptome analysis

**Figure 3:**
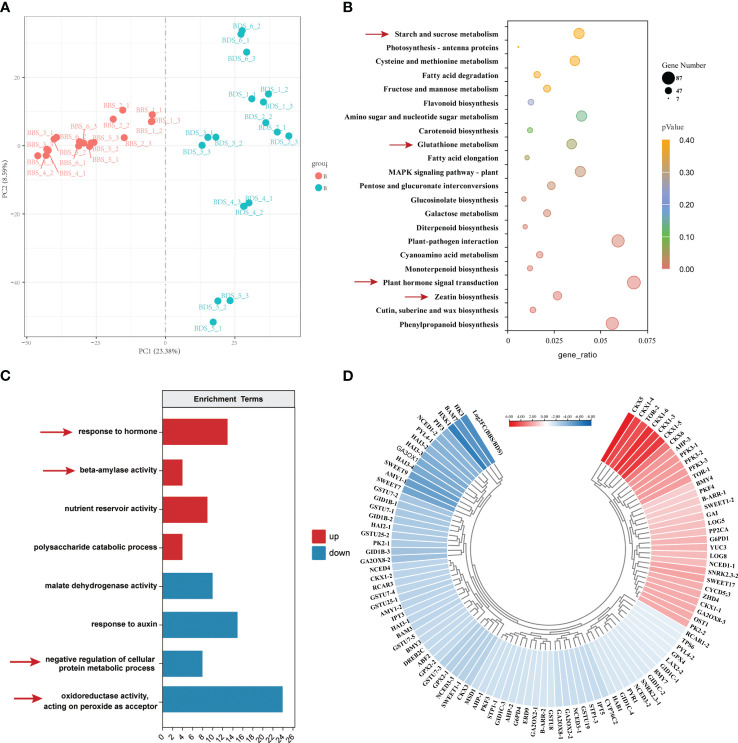
Comprehensive analysis of gene expression in the BBS *vs*. BDS: PCA results **(A)**, enriched KEGG pathways for DEGs **(B)**, enriched GO analysis for URGs and DRGs **(C)** and the identification of 103 key DEGs **(D)**.

We conducted a transcriptomic analysis to investigate the gene expression profiles of the BDS and BBS samples, and looked into the differences in the varied developmental stages. A total of 12,948 differentially expressed genes (DEGs) were identified using a stringent statistical cut–off of |log2(FoldChange)| >=1 and *P* value < 0.05. Of these DEGs, 2801 were upregulated (URGs) and 5589 were downregulated (DRG) in the comparison of BBS to BDS. The reproducibility of the results was confirmed by correlation analysis (**Supplementary Figure 2**) and PCA (**Figure 3A**), which revealed clear distinctions between the two groups. KEGG analysis was performed on the DEGs (**Figure 3B**). Specifically, ‘zeatin synthesis’, ‘plant hormone signal transduction’, ‘glutathione metabolism’ and ‘starch and sucrose metabolism’ were found to be significantly enriched. Furthermore, in the GO enrichment analysis, we observed significant enrichment (*q*<0.05) of different key terms in the URGs and DRGs (**Figure 3C**). Specifically, the URGs were found to be significantly enriched in terms related to ‘response to hormone’ and ‘beta-amylase activity’, whereas the DRGs showed significant enrichment in terms related to ‘oxidoreductase activity, acting on peroxide as acceptor’ and ‘negative regulation of cellular protein metabolic process’ separately. Based on the results of the KEGG and GO analyses, we next screened a total of 103genes (31 URGs, 72 DRGs) that are involved in various biological processes, including plant hormone synthesis and signal transduction, glucose metabolism, bud differentiation and growth, and oxidative stress. Heatmaps were generated to visualize the expression patterns of these genes (**Figure 3D**). Additional details regarding these genes can be found in **Table S2** (**Supplementary Table 2**). Specifically, genes associated with glucose metabolism and transport (*AMY*, *BMY*, *SWEET*), GA synthesis and signal transduction (*GA3OX*, *GID1B*), glutathione S–transferase (*GSTU*), and others were found to be significantly downregulated. On the other hand, genes such as the gene coding for cytokinin dehydrogenase (*CKX*) and rapamycin–coding gene (*TOR*) were significantly upregulated. To further validate the RNA–seq results, qRT–PCR was performed on 25 DEGs selected randomly from numerous significantly expressed genes. The results showed that the overall trends of 23 DEGs were in accordance with RNA–seq analysis, thereby supporting their reliability (**Supplementary Figure 3**).

### 3.3 Metabolome analysis

**Figure 4:**
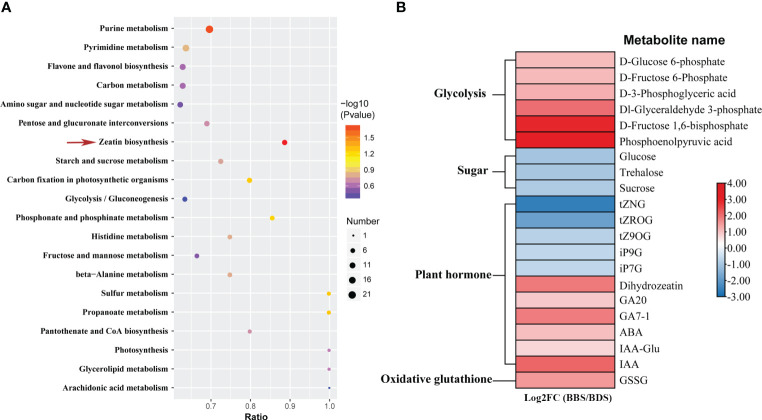
Enriched KEGG pathways for DEMs **(A)** and the identification of key DEMs **(B)**.

The process of univariate scaling was applied to the peaks extracted from the test and quality control samples, followed by principal component analysis. Outlier samples were removed, and the relationship between the remaining samples was further visualized (**Supplementary Figure 4**), which demonstrated distinct clustering of samples from two groups. Subsequently, 585 significantly differentially expressed metabolites (DEMs) were distinguished between the two groups (**Supplementary Table 3**). Then, the DEMs were assigned to 43 pathways based on KEGG analysis. Interestingly, the KEGG zeatin biosynthesis pathway was also significantly enriched for differentially accumulated metabolites same as finding in RNA-Seq analysis (**Figure 4A**). In this study, plant hormones and sugar–related metabolites were marked out (**Figure 4B**). Metabolites associated with plant hormones, including IAA, GA7–1, GA20 and ABA, all exhibited significantly increased levels in the BBS. Interestingly, significant decreases in the levels of glycosylated CKs metabolites was observed in the BBS, including for trans–zeatin–o–glucoside riboside (tZROG), trans–zeatin N–glucoside (tZNG), isopentenyladenine–9–glucoside (iP9G), trans–zeatin 9–O–glucoside (tZ9OG) and isopentenyladenine–9–glucoside (iP7G). Moreover, we also noted that the majority of glycolysis–related metabolites had significantly increased levels compared to the BDS. However, metabolites belonging to the sugar class, such as glucose, sucrose and trehalose, had decreased levels in the BBS. In addition, increased level of oxidized glutathione (GSSG) in the BBS was observed.

### 3.4 Association analysis and key DEGs identification in bud sprouting stages

**Figure 5:**
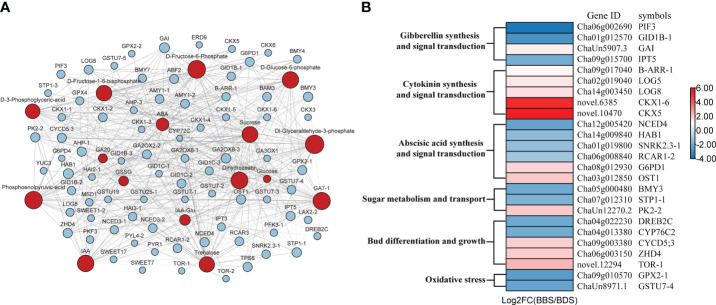
Gene–metabolite association network diagram **(A)** and Genes involved in bud dormancy transition and sprouting **(B)**. Blue squares represent genes; Red circles represent metabolites; Dashed lines represent negative correlations and solid lines represent positive correlations.

We conducted a comprehensive analysis of key genes and metabolites identified in our experiment. For each combination of genes and metabolites, we calculated the correlation coefficient between them (**Supplementary Table 4**), and only those with a Pearson coefficient greater than 0.6 were visualized (**Figure 5A**). Based on the association analysis results, we screened 25 genes markedly associated with bud sprouting stats variation in tea plants (**Figure 5B**). Our analysis revealed that the most relevant gene associated with trehalose and sucrose metabolites was *BMY3*, respectively. *ZHD4* was found to be the most relevant gene among glycolytic–related metabolites, such as D–fructose–6–phosphate and D–glucose–6–phosphate. Interestingly, we observed a positive correlation between most genes and trehalose, while there was a negative correlation between genes and glucose.

### 3.5 Antioxidant system detection in different bud sprouting stages

**Figure 6:**
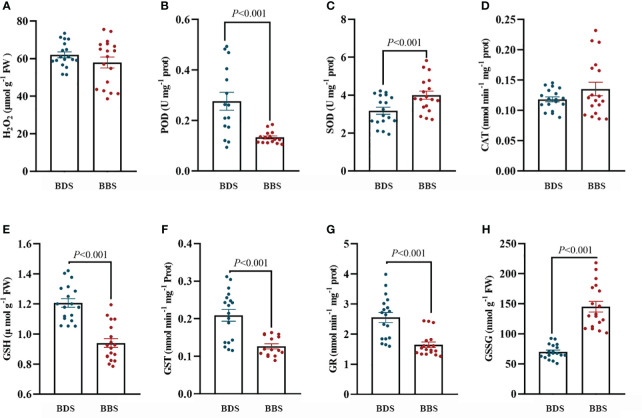
Assessment of the antioxidant system: levels of H_2_O_2_ **(A)**, POD **(B)**, SOD **(C)**, CAT **(D)**, GSH **(E)**, GST **(F)**, GR **(G)**, and GSSG **(H)**. The data are presented as the mean ± SE (Student’s *t* test), and the units of measurement for ‘U’ are per milligram of protein, while ‘Fresh weight’ (FW) is measured in micromoles or nanomoles per gram of fresh weight.

ROS production and scavenging have been shown to play a role in the transition of bud dormancy (Shangguan et al., 2020). In our experiments, we observed significant upregulation of genes associated with oxidative stress indifferent bud sprouting stages. To assess the internal antioxidant system at these distinct stages, we measured the activity levels of conventional antioxidant enzymes (CAT, POD, SOD) as well as the concentration of H_2_O_2_. Our results showed that the CAT and SOD activity levels were significantly higher in the BBS. Conversely, there were no significant differences in POD activity and H_2_O_2_ concentration observed among these stages (**Figures 6A–D**). Furthermore, a significant downregulation of the glutathione metabolic pathway and a large downregulation of glutathione transferase were observed. To investigate these findings further, the activity of GST and GR, as well as the content of GSSG and GSH were assayed. Our results showed a significant decrease in GST and GR activity, as well as a significant increase in GSSG levels and a decrease in GSH levels in the BBS (**Figures 6E–H**).

### 3.6 T6P and sugar content in different bud sprouting stages

**Figure 7:**
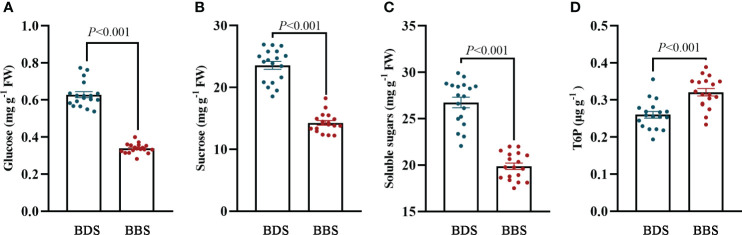
Analysis of sugar contents: levels of glucose **(A)**, sucrose **(B)**, soluble sugars **(C)**, and T6P **(D)**. The data are presented as the mean ± SE (Student’s *t* test).

The successful sprouting of buds in woody perennials in the spring is influenced by the availability of stored sugars. The regulation of this process is thought to be modulated by trehalose 6–phosphate (T6P), which plays an important role in plant growth and development (Wingler and Henriques, 2022). To further determine the change in sugar content in different bud sprouting stages, the contents of glucose, sucrose, soluble sugar and T6P were further determined. The results showed that the levels of sucrose, glucose, and soluble sugars were all decreased in the BBS, while the level of T6P was increased in the BBS (**Figures 7A–D**), indicating its potential role in regulating the sprouting process.

### 3.7 Plant hormone levels in different bud sprouting stages

**Figure 8:**
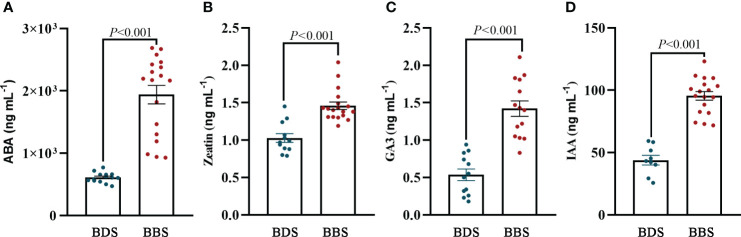
Quantification of plant hormones: levels of ABA **(A)**, zeatin **(B)**, GA3 **(C)**, and IAA **(D)**. The data are presented as the mean ± SE (Student’s *t* test).

To accurately determine the actual concentration of plant hormones in different bud sprouting stages, we determined the contents of IAA, ABA, Zeatin, and GA3 hormones in these stages. The results revealed that the levels of these four hormones were significantly higher in the BBS than in the BDS (**Figures 8A–D**). This finding is consistent with the results of the metabolome analysis.

### 3.8 Expression changes of key genes during bud dormancy to sprouting transition

**Figure 9:**
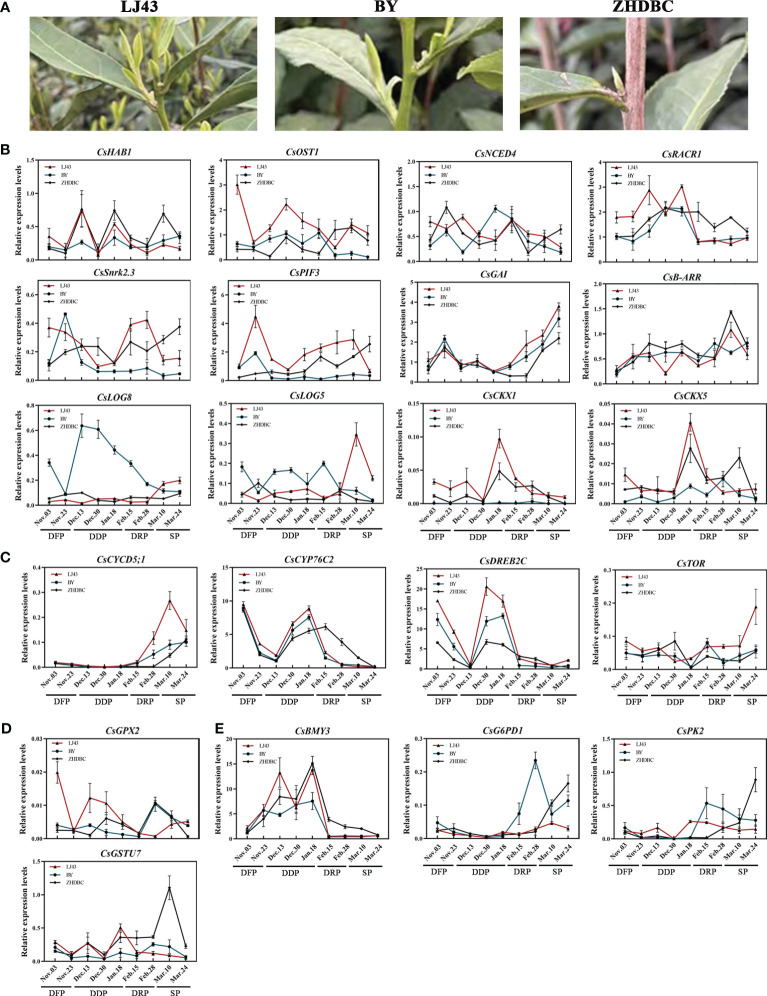
Bud phenotypes of three cultivars (LJ43, BY, ZHDBC) under Mar 24; LJ43 was at the sprouting stage, BY was at the dormancy release stage and ZHDBC was at the dormancy stage **(A)**. Expression changes of the genes related to plant hormone signalling synthesis and transport **(B)**, bud differentiation and growth **(C)**, oxidative stress **(D)** and sugar metabolism and transport **(E)** during the transition from bud dormancy to sprouting in three cultivars.

**Figure 10:**
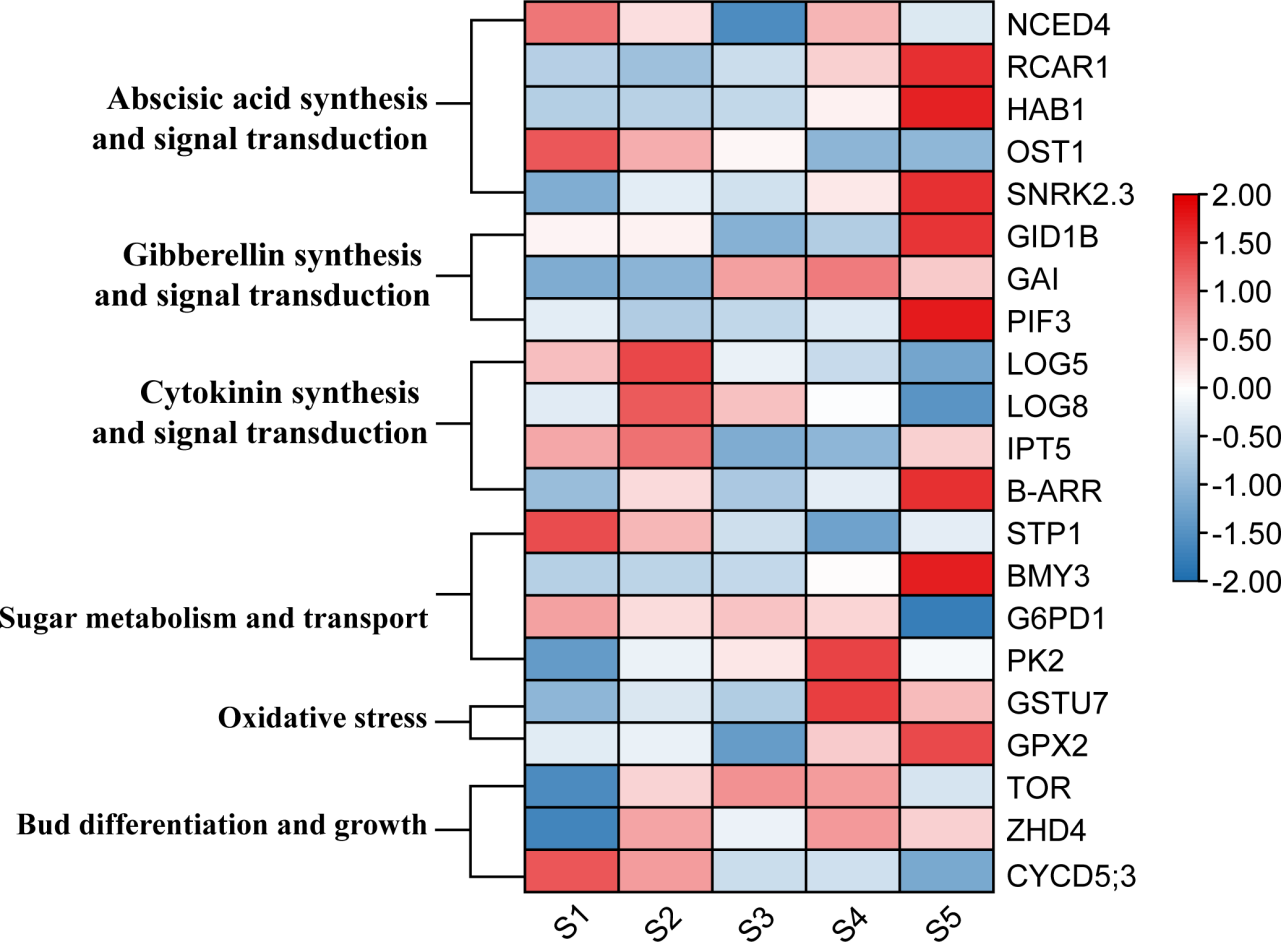
Key gene expression profiling in different bud sprouting phases of LJ43.

To comprehensively study the expression changes of the key genes during the detailed transition from dormancy to sprouting, an investigation was conducted on the changes in gene expression profiles from dormancy formation period to bud sprouting period (**Figure 5B**). Three tea cultivars with distinct phenological periods were selected for expression analyses. Particularly, on March 24, different sprouting status were observed: LJ43 was at the one bud and a leaf state, BY was at the dormancy release state and ZHDBC was at the dormancy state (**Figure 9A**). The results showed that the expression levels of genes related to plant hormone signalling transport, including CKs synthesis (*CsLOGs*) and signal transduction (*CsB–ARR*), DELLA (*CsGAI*) protein were upregulated during the bud break period (**Figure 9B**), while ABA signal transduction, *CsCYP76C2*, and *CsDREB2C* were upregulated in the bud dormancy stage. Meanwhile, the genes related to sugar transport (*CsSWEET17*), glucose metabolism (*CsBMY3*) and oxidative stress response (*CsGSTU7* and *CsGPX2*) were upregulated in the dormancy stage (**Figures 9D, E**). Otherwise, genes related to bud division and differentiation (*CsCYCD5;3*, *CsTOR*) were upregulated in the bud break period (**Figure 9C**). To further investigate the role of these genes in bud sprouting, transcriptional analysis was performed on buds collected from the five sprouting stages of the tea cultivar LJ43. Transcription expression profiling (**Figure 10**) revealed high expression levels of bud differentiation–related genes (*ZHD4*, *CYCD5;3*, and CKs synthesis (*LOGs* and *IPT5*) in the early stage of sprouting, with a gradual decrease during subsequent stages. However, ABA signal transduction components (*Snrk2.3* and *HAB1*), GA signal transduction components (*GID1B*, *PIF3*), bud growth (*CYP76C2*, *DREB2C*) and sugar transport and synthesis genes *BMY3* remained low before the S5 stage.

## 4 Discussion

Tea bud sprouting typically takes place in response to favorable environmental conditions and is subject to complex regulation by a variety of factors. In this study, the tea buds in different bud sprouting stages were selected to investigate the internal mechanism in bud break regulation in spring. The daily temperature was continually recorded to evaluate the suitability of the environmental conditions for tea plant sprouting. It was found that the majority of days had a temperature above 13°C, which is considered as a threshold value for tea plant growth (Barua, 1969). Despite these favorable conditions, differences in the sprouting of tea cultivars were observed. Histological analysis of these lines showed that break bud had a higher degree of development, with more developed young leaves and looser tissue structure, indicating stronger growth potential compared to the buds from the dormancy bud. Moreover, PCA of these line’s expression profile also revealed significant differences between the different bud sprouting stages. Tea bud sprouting regulation have been focused in our previous studies (Yue et al., 2018; Hao et al., 2019), but the overall level of genes and metabolism affecting tea plant bud sprouting remains unclear. Therefore, metabolic and transcriptional analyses were performed on these buds to identify key genes and metabolites involved in bud sprouting, which allowed for a more complete understanding of the process. The analysis initially identified key genes and metabolites involved in bud sprouting, and subsequent gene expression profiles and metabolite quantification levels confirmed the reliability of the results. Our research provides new insights into the underlying mechanisms that govern the sprouting of tea plant buds.

### 4.1 Plant hormones signals in regulating tea bud sprouting

Plant hormones play a crucial role in regulating various aspects of plant growth, development, and stress response, including seed germination and plant bud growth (Kavi Kishor et al., 2022). The presence of specific hormones, such as GA and ABA, triggers the breaking of seed dormancy and the initiation of bud sprouting. GA and ABA play opposite roles in triggering the breaking of seed dormancy and the onset of bud sprouting. GA promotes seed germination by breaking seed dormancy, while ABA inhibits germination by keeping seeds dormant and preventing seed water absorption (Shu et al., 2016). Additionally, ABA is recognized as a general inhibitor of plant branching due to its ability to reduce BR and GA levels and increase IAA levels in axillary buds (Yao and Finlayson, 2015). Under low red light (R)/far–red light (FR) conditions, ABA is produced in upper leaves and transported to lower axillary buds via stem segments, thereby inhibiting axillary bud sprouting. This process is mediated by *PP2C* and *SnRK2* family members in the ABA signalling pathway (Reddy et al., 2013). On the other hand, GA synthetase and GA–inducible 1,3–β–glucanases are involved in the degradation of plasmodesmata callose during bud dormancy release, thus promoting bud sprouting (Rinne et al., 2016). Previous research has shown that the application of exogenous GA3 can prompt the release of dormant buds in tea plants. However, GA can either promote or repress axillary bud formation, depending on the concentration and timing of application (Ni et al., 2017; Zhang et al., 2020). In contrast to the effect of ABA, CKs treatment has been observed to expedite the development of buds in certain plant species, which may potentially be attributed to the promotion of gene expression related to the cell cycle, carbohydrate metabolism, and signal transduction (Ni et al., 2017; Zhao et al., 2020).

In this study, plant hormone signalling pathways were substantially enriched in KEGG analysis, and significant differences in expression levels were observed for some key genes associated with these processes. Specifically, a significant increase in ABA content was observed despite the downregulation of ABA signal transduction–related genes at the bud sprouting stage. Usually there is a high level of ABA content in buds to maintain plant dormancy during endodormancy phase, followed by a decrease of ABA content in bud sprouting stage (Pan et al., 2021). Particularly, previous study showed that the sprouting of tea plants was related to the ratio of hormones such as zeatin/ABA, rather than the absolute content of ABA (Hao et al., 2018b). Similarly, a significant increase in zeatin content was observed during the sprouting stage in this study. It is speculated that the gene network involved in tea plant sprouting regulation is in response to hormones ratio rather than absolute content in buds. Nevertheless, a further in-depth study is required to understand the coordination mechanism of hormones in sprouting regulation. Interestingly, *CsGAI*, a DELLA protein, was observed to significantly upregulate changes during early bud sprouting. In *Arabidopsis*, axillary bud sprouting is enhanced by low concentrations of GA. The activation of axillary bud sprouting involves an activated DELLA protein that indirectly mitigates the inhibitory activity of *LAS*, which is mediated by GA (Zhang et al., 2020). A similar mechanism may be at play in promoting early bud sprouting and growth in tea plants. Thus, the interaction between DELLA protein and GA may be an important mechanism in regulating bud growth and development. Specifically, DELLA protein may act to modulate the inhibitory activity of *LAS*, allowing for the activation of axillary bud sprouting. This process is likely mediated by a complex interplay between DELLA protein and other plant hormones (Daviere and Achard, 2016).Intriguingly, zeatin synthesis was a notably enriched metabolic pathway in the bud sprouting regulation. Furthermore, the dihydrozein content and the expression of *CKX* genes and CKs–activating enzymes were significantly upregulated in bud break, along with notable changes in genes associated with the cell cycle and glucose metabolism. These findings provide additional evidence for the role of zeatin in regulating bud sprouting. As a cytokinin, zeatin plays a crucial role in axillary bud differentiation and growth (Nemchinov et al., 2016). Notably, there is a significant downregulation of zeatin glycosylated metabolites, which are typically regarded as inactive CKs. Bud break may be producing more free zeatin, which is more biologically active and thus may have a greater impact on bud differentiation and growth. The observation of a significant upregulation of *CKX* may appear contradictory to previous reports. However, this finding may be explained by Schmülling’s research, which suggests that increased CKs activity is often accompanied by increased CKX activity, which helps to maintain CKs homeostasis (Motyka et al., 1996). In addition to the upregulation of CKs synthesis and signal transduction, an increase in IAA content in the bud break stage was also observed. Elevated levels of CKs promote the transport of auxin from axillary buds to stem segments or increase local auxin synthesis. This activation of axillary bud growth suggests that the transportation of auxins from axillary buds to stems is a crucial factor in the process (Tan et al., 2019). On the other hand, the IAA content increase in the bud break stage might also be due to enhanced *de novo* synthesis (Shimizu-Sato et al., 2009) by various enzymes, such as YUC enzymes, in the buds themselves, while auxin transport capacity increased due to upregulation of the *PIN* gene. Alterations in *PIN* gene expression and localization impact the levels of auxin, thus modulating the process of axillary bud sprouting (Balla et al., 2016). In our study, we also observed an upregulation level of the *PIN* genes on the sprouting stages although they did not reach statistical significance. On the basis of these findings, we infer that the regulation of the *PIN* genes may increase the expression level of membrane protein transporters, thereby increasing auxin transport capacity.

### 4.2 Sugar metabolism in regulating tea bud sprouting

Different sugars play distinct roles in bud sprouting and growth. Glucose has been shown to repress the expression of CKX in *Arabidopsis*, leading to increased CKs accumulation, which promotes cell division and growth (Choudhary et al., 2022). Sucrose, on the other hand, impacts multiple metabolic pathways in axillary buds, including glycolysis, the TCA cycle, OPPP, and amino acid metabolism (Wang et al., 2022). High concentrations of sucrose have been shown to counteract the inhibitory effects of auxin on axillary buds and stimulate their growth while promoting bud dormancy by rapid transport from the apical meristem (Barbier et al., 2015). During bud development, the sugar content and metabolic pathways within the bud change dynamically to meet the bud’s changing energy needs. Dormant buds need to store sugar to break dormancy and support growth and development once conditions become favorable (Sheikh et al., 2022). In this study, we observed a significant reduction in glucose, sucrose, trehalose, and soluble sugar content in the bud break stage through metabolic analysis. Genes associated with glucose metabolism and transport were significantly upregulated during bud dormancy, suggesting that the bud is actively preparing for growth and development once conditions become favorable. Additionally, a considerable number of genes and metabolites associated with glycolysis were upregulated, indicating that bud energy production and synthetic capacity are enhanced during bud development. As discussed above, the bud acts as a sugar reservoir to accumulate sufficient energy to support vital metabolic processes during the dormant phase. With the onset of spring and the corresponding rise in temperature, the metabolic activity of the bud increases, and the stored sugars are utilized as a primary energy source. This event triggers the activation of the glycolytic pathway, which is responsible for energy production through the breakdown of glucose. This pathway also enhances the bud’s synthetic capacity, allowing for the production of necessary components for growth and development.

### 4.3 T6P and TOR kinase in regulating tea bud growth and development

T6P and TOR kinase are two critical regulators of sugar metabolism and growth in plants. T6P has been shown to affect shoot branching and bud growth, with low levels leading to decreased bud growth and high levels promoting branch formation (Ponnu et al., 2011; Fichtner et al., 2021). Moreover, T6P regulates the expression of SNF1–related protein kinase 1 (*SnRK1*) and sucrose levels (Fedosejevs et al., 2018). On the other hand, TOR kinase plays a central role in integrating various signals that modulate growth and metabolic responses (Xiong and Sheen, 2015). Various compounds, including sugars, nitrates, amino acids, and ROP/RAC small GTPases, have been suggested to effectively activate TOR kinase activity (Xu et al., 2014; Xiong and Sheen, 2015; Tulin et al., 2021). Glycolysis and mitochondrial electron transfer chain (ETC)–mediated glucose metabolism, downstream of the TOR kinase, significantly influence its activity, and the inhibition of these processes can effectively suppress the TOR kinase (Xiong et al., 2013). In this study, GO analyses of the BBS revealed some processes that could be potentially associated with the mechanism of action of the TOR kinase, including ‘DNA replication’, ‘cellular response to DNA damage stimulus’, and ‘cell cycle’ (Magyar et al., 2012). Furthermore, the TOR kinase gene exhibited increased expression in the BBS and upregulation during bud sprouting, while T6P content was also observed to be upregulated in the BBS. Therefore, TOR kinase and T6P may cooperatively function upstream of plant hormones, influence glucose metabolism, and regulate bud growth and development. However, further studies are needed to elucidate the specific molecular mechanisms underlying these processes.

### 4.4 Glutathione transferase and oxidative stress in tea bud break from dormancy

ROS tend to accumulate during dormancy due to cold stress and reduced metabolic activity (Sapkota et al., 2021). The *GST* family comprises numerous members and serves as a significant plant defense mechanism (Estévez and Hernández, 2020). Recently, Cao identified and analysed 88 *GST* genes in tea plants and found that they exhibited potential functions in response to stress (Cao et al., 2022). However, the role of *GST* during bud dormancy in tea plants needs to be further investigated. In our transcriptome data, a substantial number of *GSTs* exhibited upregulation in the dormancy stages. Correspondingly, their enzymatic activity was also enhanced. This finding is consistent with previous research that has reported an increase in *GST* activity and expression levels during dormancy induction and a decrease during dormancy release in *Castanea crenata* buds (Nomura et al., 2007). Moreover, glutathione transferase is able to bind glutathione to other compounds to clear reactive oxygen species, and therefore, the common forms of glutathione and glutathione reductase were measured. The results showed that there was a significant downregulation of GSSG and a concomitant upregulation of GSH in the bud break stages, indicating a decrease in the level of oxidative stress in dormant buds. In addition, GR exhibited downregulated expression, which further indicates the reduction in oxidative stress in dormant buds (Zanol et al., 2007). The elevation of the GSH/GSSG ratio signifies a reduced level of oxidative stress in dormant buds, providing further evidence of the increased antioxidant capacity of dormant buds. Furthermore, the effective removal of ROS and prevention of oxidative damage in buds is largely attributed to the pivotal function of diverse antioxidant enzymes. In the dormancy bud stages, the transcriptomic analysis revealed the upregulation of genes encoding *GPX* and *MSD* and notably elevated levels of POD that exhibited a significant increase in the subsequent enzyme activity assays. These results indicate an enhanced antioxidant capacity of dormant buds, highlight the importance of the antioxidant defense mechanisms of tea buds during dormancy.

### 4.5 Major pathway governing bud sprouting in tea plants

**Figure 11:**
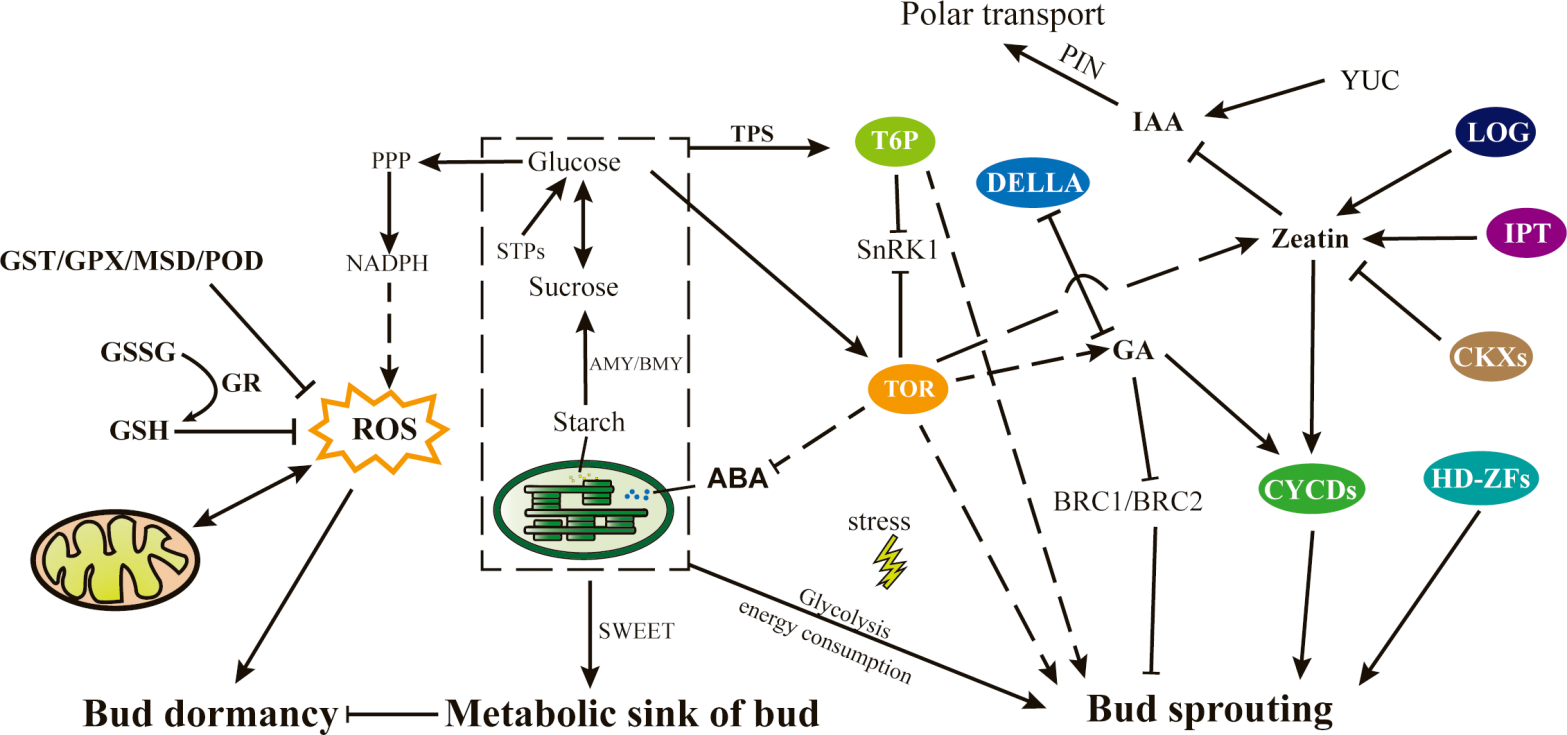
Proposed model of the mechanism of bud sprouting in tea plant. Directional relationships are represented by arrows, denoting direct effects, while dotted lines signify indirect effects. ABA, Abscisic acid; AMY/BMY, α/β amylase; *BRC*, Branched; CKX, Cytokinin dehydrogenases; *CYCDs*, Cyclin Ds; GA, Gibberellic acid; GSH, Glutathione; GSSG, Oxidized glutathione; GST, Glutathione S-transferase; GPX, Glutathione peroxidase; GR, Glutathione reductase; *HD-ZFs*, Homeodomain-leucine zipper transcription factors; IAA, Indole-3-acetic acid; IPT, Isopentenyl transferases; LOG, Lonely guy; MSD, Methionine sulfoxide decarboxylase; NADPH, Nicotinamide adenine dinucleotide phosphate; PIN, PIN-formed; PPP, Pentose phosphate pathway; ROS, reactive oxygen species; SnRK1, SNF1-related protein kinase1; STPs, Sucrose transport proteins; TOR, Target of rapamycin; TPS, Trehalose-6-phosphate synthase; T6P, Trehalose-6-phosphate; YUC, YUCCA.

Through the integration of our findings and previous reports, we have suggested a major pathway that governs bud sprouting in tea plants (**Figure 11**). In brief, the transition from bud dormancy to bud break is intricately linked to various factors such as carbohydrate content, energy metabolism, oxidative stress, and antioxidant capacity within the tea buds. During the dormant phase, buds serve as a reservoir for accumulating adequate energy to sustain crucial metabolic processes while simultaneously upregulating the expression of antioxidant enzymes to counteract oxidative stress. Maintaining an appropriate level of oxidative stress within the bud is a crucial factor for the initiation of sprouting. As spring approaches, an increase in temperature prompts the activation of buds, and the partial consumption of stored sugars as an energy source also promotes the activation of glycolytic pathways, thereby enhancing their own biosynthetic capacity. Sugars are utilized to synthesize various biological macromolecules, while the remainder accumulate as a source of carbon and energy to support cell division and tissue development, thereby facilitating bud growth and development. Additionally, the rapid growth of germinated buds is accompanied by the continuous development of chloroplasts, as well as the metabolism of plant hormones such as ABA, zeatin, GA, and auxin, which collectively regulate bud growth and development via their respective signal transduction pathways. Several key factors, including TOR kinase, T6P, transcription factors of the cyclin and HD–ZF families, and enhanced expression of *LOG*, *GAI* and *IPT* genes, contribute to this complex regulatory process.

## 5 Conclusions

In this study, tea buds in different sprouting states were selected to investigate the molecular basis of tea bud sprouting and identify key factors involved in the process. Transcriptional and metabolic analyses identified key genes and metabolites involved in bud sprouting, and a comprehensive pathway that governs bud sprouting in tea plants was suggested. This pathway is intricately linked to various factors such as carbohydrate content, energy metabolism, oxidative stress, and antioxidant capacity within the buds. Notably, the metabolism of soluble sugars, such as glucose and sucrose, as well as the effective clearance of reactive oxygen species, have emerged as pivotal elements preceding bud sprouting. Additionally, plant hormones, especially zeatin, was unveiled the central role in orchestrating bud growth. And regulatory proteins like CYCDs and TOR contribute to this complex regulatory process. Overall, this study provides insights into the complex mechanisms underlying tea plant bud sprouting.

## Data availability statement

The data presented in the study are deposited in the NCBI Sequence Read Archive (SRA) experiment SRX20411675 repository, accession number SRR24631004 to SRR24631050.

## Author contributions

JT: Data curation, Formal Analysis, Investigation, Visualization, Writing – original draft. YC: Formal Analysis, Investigation, Visualization, Writing – original draft. CH: Formal Analysis, Investigation, Writing – original draft. CL: Investigation, Writing – original draft. YF: Methodology, Writing – review & editing. HW: Investigation, Writing – original draft. CD: Supervision, Writing – review & editing. NL: Supervision, Writing – review & editing. LW: Supervision, Writing – review & editing. JZ: Supervision, Writing – review & editing. YY: Supervision, Writing – review & editing. XH: Funding acquisition, Supervision, Writing – review & editing. XW: Supervision, Writing – review & editing.

## Supplementary material

Supplementary Figure 1 Temperature records during the period of March 2022 to April 2022.

Supplementary Figure 2 Sample correlation analysis in different bud sprouting stages.

Supplementary Figure 3 Validation of transcriptome genes by qRT–PCR.

Supplementary Figure 4 PCA of the metabolome in the BBS and BDS.

Supplementary Table 1 Information on qRT–PCR primer design.

Supplementary Table 2 Key DEGs information related to bud dormancy and sprouting.

Supplementary Table 3 Differential expression of metabolites identified in different bud sprouting stages.

Supplementary Table 4 Information on gene–metabolite association analysis.
