# Supplementary figures and images for: Uncovering the complex regulatory network of spring bud sprouting in tea plants: insights from metabolic, hormonal, and oxidative stress pathways
Source: Front Plant Sci. 2023 Oct 23;14:1263606. doi: 10.3389/fpls.2023.1263606 (PMC10627156; doi:10.3389/fpls.2023.1263606)

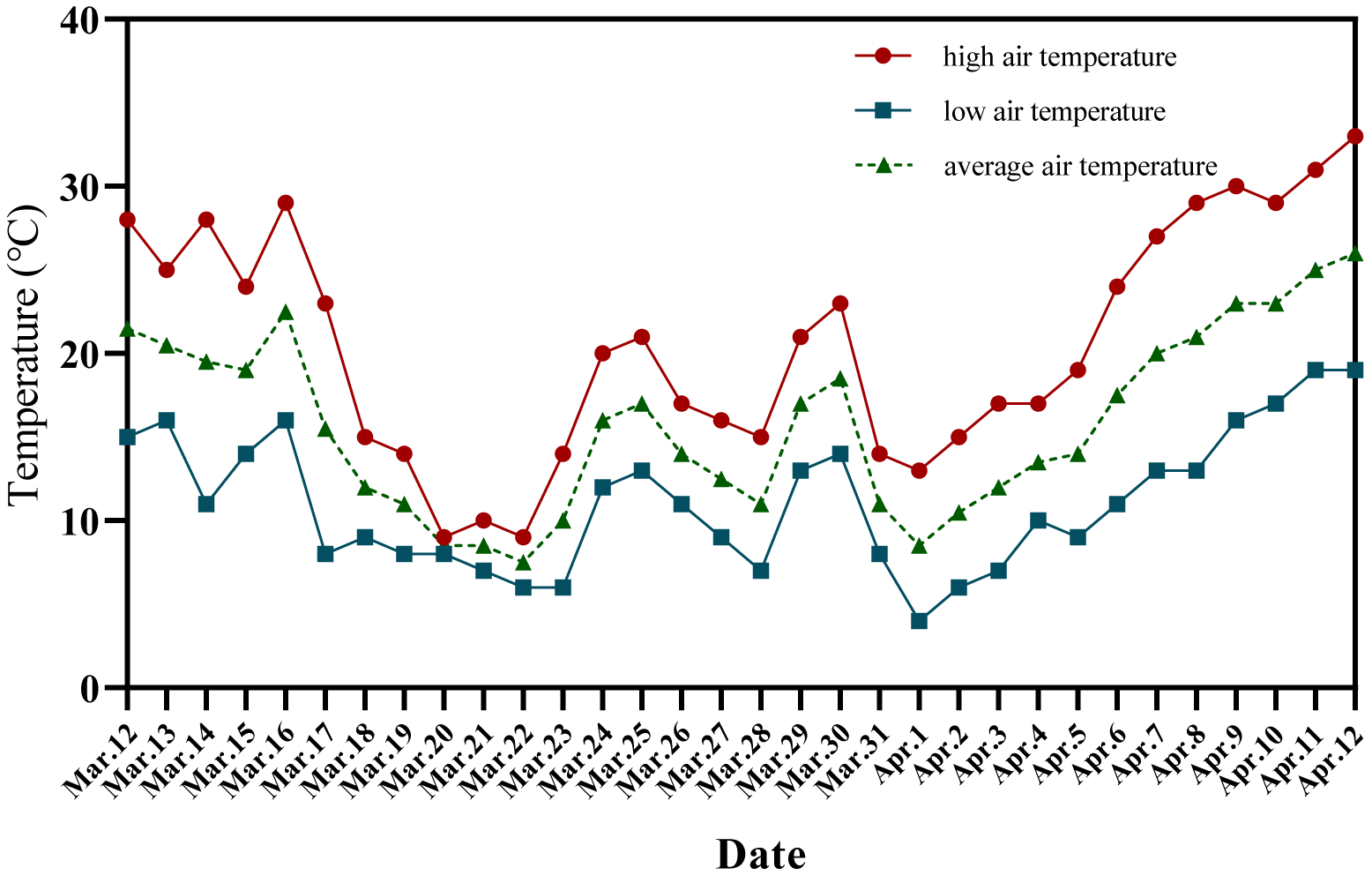

Supplement: Supplementary Figure 1 — Temperature records during the period of March 2022 to April 2022. [file Image_1.tif]

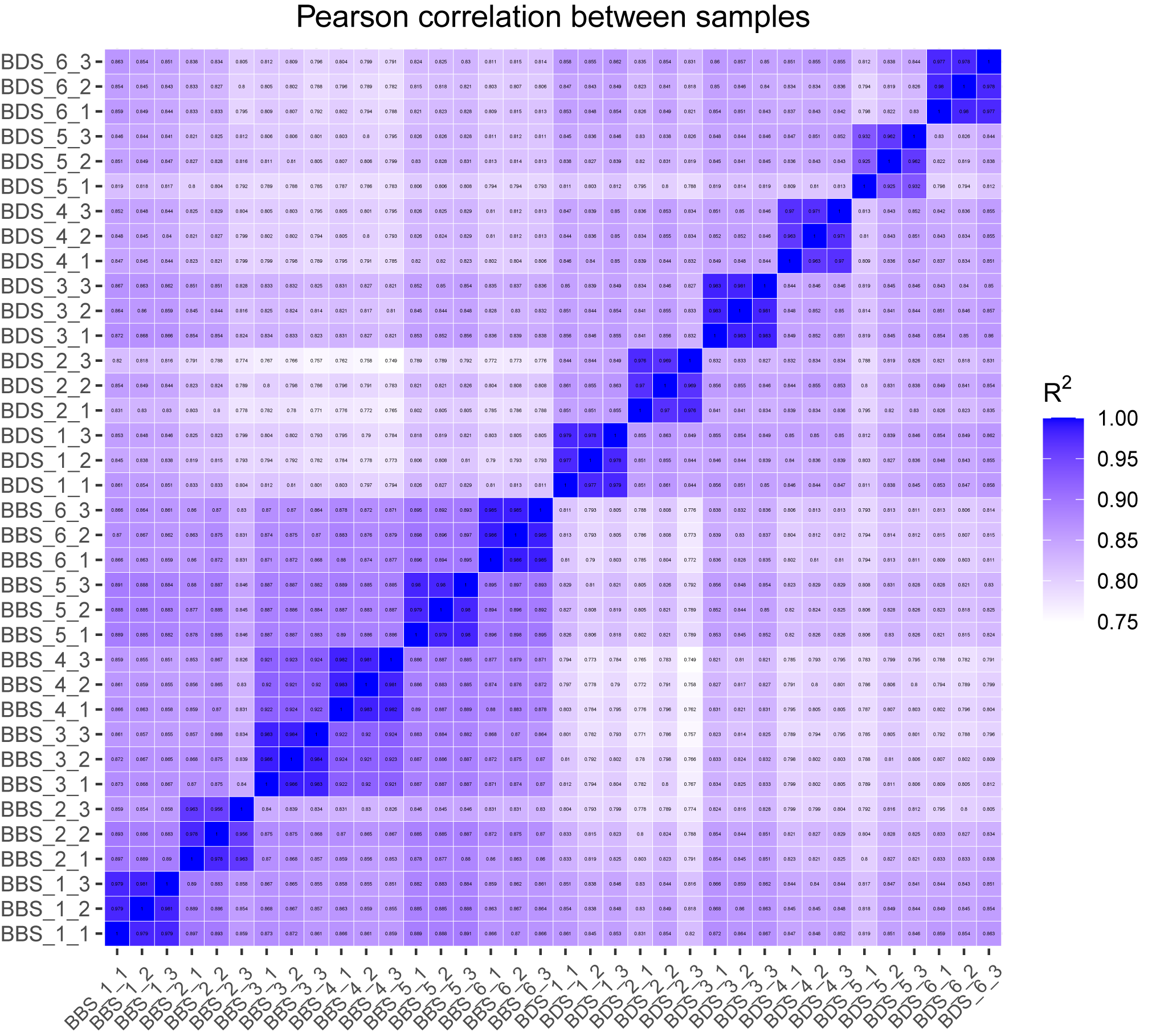

Supplement: Supplementary Figure 2 — Sample correlation analysis in different bud sprouting stages. [file Image_2.tif]

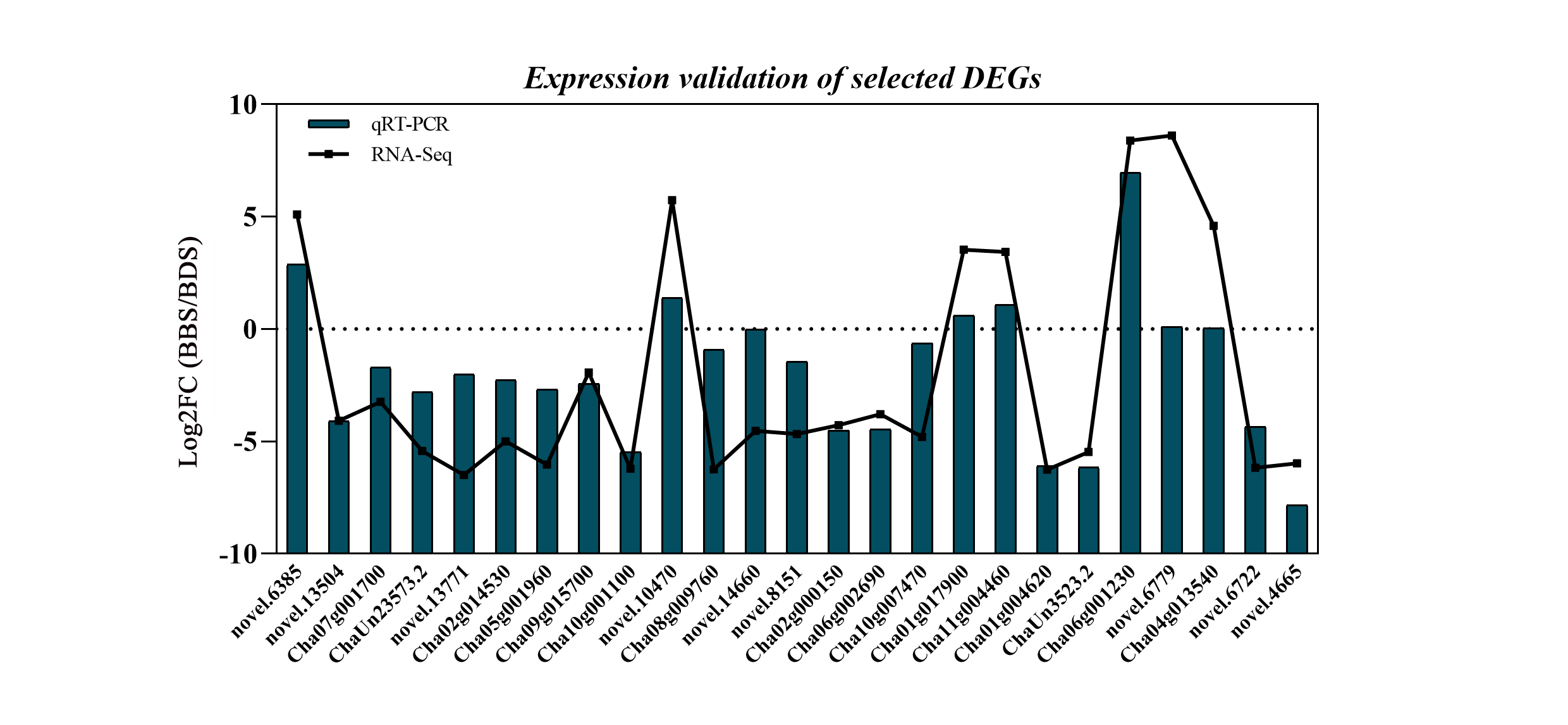

Supplement: Supplementary Figure 3 — Validation of transcriptome genes by qRT–PCR. [file Image_3.tif]

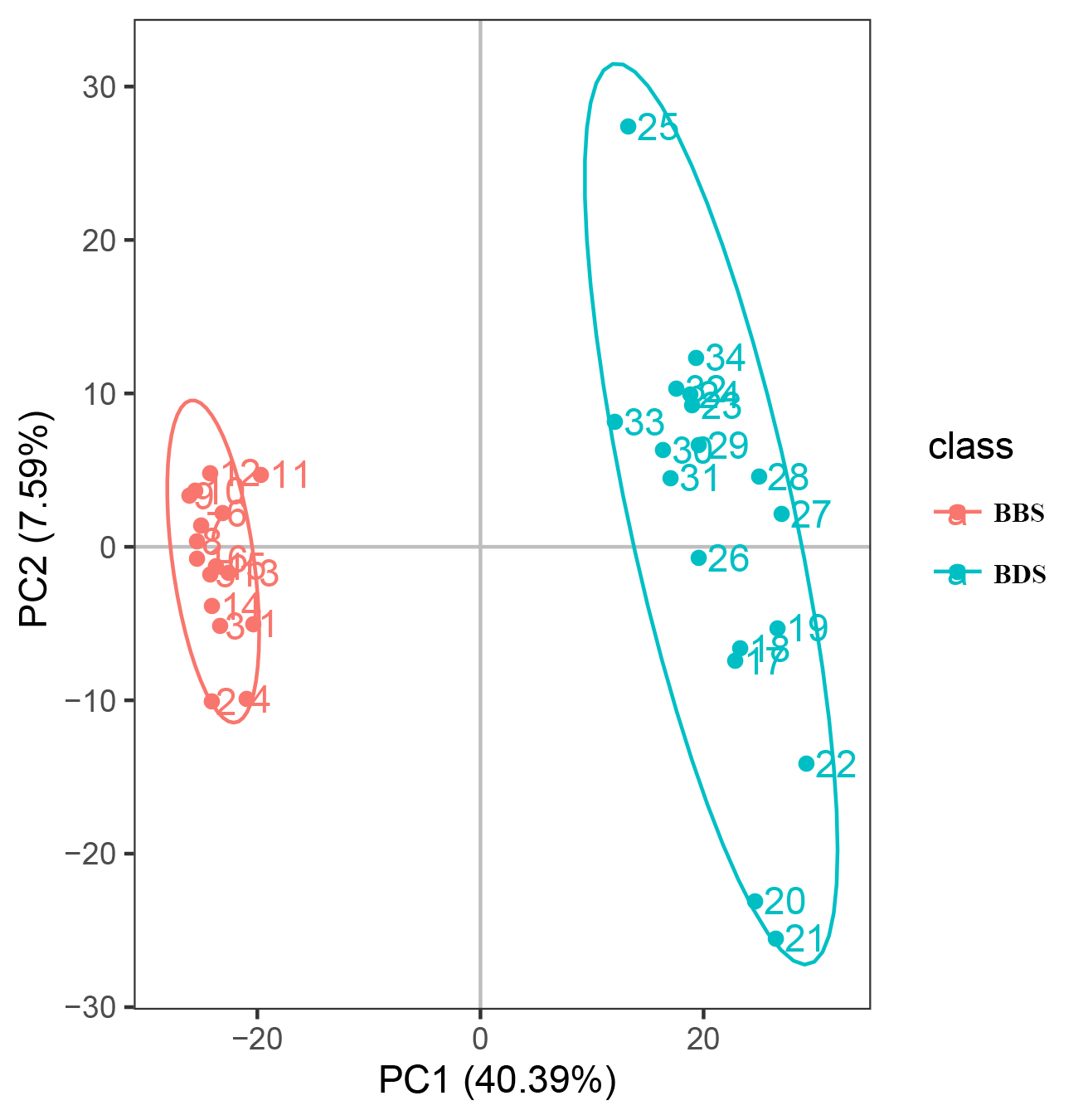

Supplement: Supplementary Figure 4 — PCA of the metabolome in the BBS and BDS. [file Image_4.tif]
